# Supplementary material for: Cell Assembly Dynamics of Sparsely-Connected Inhibitory Networks: A Simple Model for the Collective Activity of Striatal Projection Neurons
Source: PLoS Comput Biol. 2016 Feb 25;12(2):e1004778. doi: 10.1371/journal.pcbi.1004778 (PMC4767417; doi:10.1371/journal.pcbi.1004778)
Supplement: S1 Text — (PDF) [file pcbi.1004778.s001.pdf]

S1 TEXT

Silent Neurons

We define a neuron as silent, if it does not emit more than S_Θ spikes within the system evolution, which we typically take as the time taken for the network to evolve through to 10^7 spikes. In particular, in S 1 (a,b) Figs we report the fraction of active neurons n^* versus the synaptic strength for two parameter settings and for several values of the considered threshold, namely $0 \leq S_\Theta \leq 100$. In practice, we observe that neither the minimal value of n^* nor the value for which the minimum is reached, appears to strongly depend on the chosen threshold, thus demonstrating the robustness of the results that we present through the article.

Mechanisms for the resurgence of silent neurons

In what follows we report the neuronal distributions of the average inter-spike intervals $\overline{ISI}$, of the corresponding CV, of the associated average effective synaptic input $\overline{W}_i \equiv I_i - g\overline{E}_i$ and standard deviation $\sigma(W_i)$. In particular, we consider these distributions for two different stimuli dispersion (namely, $\Delta V = 5$ mV and 1mV) as well as for two synaptic strengths (namely, $g \simeq g_{min}$ and $g >> g_{min}$).

For $\Delta V = 5$ mV ($\Delta V = 1$ mV) we examine two synaptic strengths, one in proximity of the minimum g_{min} of n^*, where almost 50 % of neurons are active, and one for which almost all the neurons are active again, namely $g = 4$ and 10 ($g = 1$ and 4). Let us first consider the distribution of the average $\overline{ISI}$ of the single cell reported in S 2(a,d) Figs. At small g the distributions reveal a clear peak at some low $\overline{ISI}$ plus a long tail. In correspondence of this coupling the distribution of CV is clearly bimodal as shown in S 2(b,e) Figs with peaks around zero and one, thus indicating that the neurons associated to the peak in $P(\overline{ISI})$ are firing in a regular fashion, while the neurons in the tail of $P(\overline{ISI})$ contributes to the second peak in $P(CV)$ around $CV \simeq 1$. Furthermore, by examining the distributions of the average effective input $\overline{W}_i$ perceived by each single cell, the PDF for $g \simeq g_{min}$ has a peak in proximity the threshold value V_{th}.

We can conclude that the neurons contributing to the main peaks in $P(\overline{ISI})$ and $P(CV)$ for $g \simeq g_{min}$ are the *winners*, which fire faster than the others and almost periodically, thus suggesting that they are not particularly influenced by the other neurons in the network. Moreover, they correspond to the neurons which are on average above threshold, as shown in S 2(c,f) Figs. The neurons contributing to the second maximum in $P(CV)$ and to the tail of $P(\overline{ISI})$ are instead slow neurons whose activity is strongly depressed by the winners and they are neurons around, or just below threshold, in S 2(c,f) Figs.

As one can appreciate from S 2(a,d) Figs the $P(\overline{ISI})$ is completely modified at large g. In such a case, a broad peak is present extending over two orders of magnitude. In this regime the majority of the cells are on average below-threshold, as it can be appreciated by the corresponding $P(\overline{W}_i)$, reported in S 2(c,f) Figs as red empty squares, which reveal an almost Gaussian shape centered well below threshold. Therefore we are now in a situation where all neurons are active, but the majority are activated due to the fluctuations in the input and they are no more tonically firing. The fact that now the activity is mostly fluctuation driven, is reflected also in the CV distributions, which are now centered well above one.

The reported results clearly show that for wider dispersion of the I_i, as measured by ΔV, a greater lateral inhibition is required to observe similar effects.

Linear stability analysis

One of the questions that we would like to address is whether the existence of a bursting correlated activity is related to linear stability properties of the network or not. To characterize these properties, we calculate the maximal Lyapunov exponent (LE) λ for the parameters examined in the text. In order to compute the LE we derive from Eq. (3) (main text) its linearization, which describes the evolution of infinitesimal perturbations in the reference orbits,

this reads as:

$$\delta E_i(n+1) = \mathrm{e}^{-\alpha\tau(n)}\left[\delta E_i(n) + \tau(n)\delta P_i(n)\right] - \mathrm{e}^{-\alpha\tau(n)}\left[\alpha E_i(n) + (\alpha\tau(n)-1)P_i(n)\right]\delta\tau(n)\,, \tag{S1}$$

$$\delta P_i(n+1) = \mathrm{e}^{-\alpha\tau(n)}\left[\delta P_i(n) - \alpha P_i(n)\delta\tau(n)\right]\,, \tag{S2}$$

$$\delta v_i(n+1) = \mathrm{e}^{-\tau(n)}\left[\delta v_i(n) + (a - v_i(n))\delta\tau(n)\right] + g\delta H_i(n) \quad i = 1,\ldots,N \quad ; \quad \delta v_m(n+1) \equiv 0\,. \tag{S3}$$

The boundary condition $\delta v_m(n+1) \equiv 0$ is a consequence of the event driven evolution. The expression of $\delta\tau(n)$ can be computed by differentiating Eqs. (4) and (5) (in main text), namely

$$\delta\tau(n) = \tau_v \delta v_m(n) + \tau_E \delta E_m(n) + \tau_P \delta P_m(n)\,, \tag{S4}$$

where

$$\tau_v := \frac{\partial \tau}{\partial v_m} \quad , \quad \tau_E := \frac{\partial \tau}{\partial E_m} \quad , \quad \tau_P := \frac{\partial \tau}{\partial P_m} \quad . \tag{S5}$$

The maximal LE λ is defined as the the average exponential growth rate of the infinitesimal perturbation

$$\delta = (\delta v_1 \ldots \delta v_N,\, \delta E_1 \ldots \delta E_N,\, \delta P_1 \ldots \delta P_N)$$

measured through the equation

$$\lambda = \lim_{t\to\infty} \frac{1}{t} \log \frac{\mid \delta(t) \mid}{\mid \delta_0 \mid} \quad , \tag{S6}$$

where δ_0 is the initial perturbation. The evolution of the perturbation $\delta(t)$ at the following times can be obtained by integrating S1-S3 Eqs in the tangent space in parallel with the evolution in the real space and by performing at regular time intervals the rescaling of its amplitude to avoid numerical artifacts, as detailed in [2]. A positive λ denotes a chaotic dynamics, a zero maximal LE is associated to a periodic (or quasiperiodic) orbit, and a negative one to a stable fixed point. It is important to stress that, since we are dealing with an event driven map formulation of the dynamics, the zero Lyapunov exponent which is always present for continuous time evolution and associated to the growth rate of a perturbation along the orbit, is automatically discarded. This implies that, if the evolution is stable, either a fixed point or a periodic solution, we measure in both cases a maximal LE $\lambda < 0$.

For a fixed pulse duration $\tau_\alpha = 20$ ms, the behaviour of the maximal LE λ as a function of the coupling g, for different excitability spreading ΔV, is definitely different. As shown in S3(a) Fig, for $\Delta V = 1$ mV the LE (as expected) is zero for very weakly coupled systems, then it first increases with g and reaches a maximum around $g = 2$ and then it decreases monotonically becoming negative for $g > 5$. For $\Delta V = 5$ mV, the LE is always positive and increases with g saturating at an almost constant value $\lambda \simeq 3.4$ Hz for $g \geq 6$. We are specifically interested in the conditions for which the measure Q_0 is maximized, these points are indicated in S3(a) Fig, as one can notice they correspond for both considered ΔV to positive λ.

Additionally we have analyzed the behaviour of λ as a function of τ_α by fixing g to the value that maximizes Q_0 in the previous analysis. In this case it appears that λ increases with τ_α and becomes definitely negative for sufficiently small τ_α (as shown in S3(b) Fig), in agreement with the results reported in [1, 3]. The cell assembly dynamics of our network resembles that of MSNs for large τ_α, as explained in the text, the point where Q_0 is maximal are indicated also in S3(b) Fig. These evidences seem to suggest that the striatally relevant dynamics correspond to a chaotic regime, but located in proximity of the transition between chaotic and non-chaotic evolution. The same conclusion was already reported for a rate model of the striatum in [6].

However, all this analysis and the one reported in [6] consider only infinitesimal perturbations, while it has been clearly demonstrated that for inhibitory networks finite perturbations play a fundamental role as shown in [1, 3, 4, 7]. In particular our model, even for $\lambda < 0$, can display erratic evolution almost indistinguishable from chaos due to the so-called Stable Chaos mechanism [1, 5]. This leads us to conclude that the usual Lyapunov exponent is unable to capture the degree of erratic motion present in these systems, due to the possible amplification of finite amplitude perturbations.

State Transition Matrices for different regimes

In the main text we have just reported the averaged State Transition Matrix (STM) corresponding to the consecutive presentation of two stimuli for parameters obtained by maximizing Q_0. Here we want to show how the STM is modified by considering $\tau_\alpha = 20$ ms, for which Q_0 is maximal, and for a smaller pulse duration, namely $\tau_\alpha = 2$ ms, for which the evolution of the network is seemingly Poissonian. The upper panel of S5 Fig show another realization of the network obtained for the same parameters of Fig. 5 (in main text). The lower panels correspond to $\tau_\alpha = 2$ms. The raster plots clearly show that for $\tau_\alpha = 20$ ms the network exhibits a clear patterned activity with frequent switch from an activated assembly to another, furthermore there is a low correlation between the network activities in presence of the two different stimuli. As shown in S 5(b,c) Figs. For $\tau_\alpha = 2$ ms the system presents much less variability. While it is still capable of discriminating between two different stimuli, now the system fails in revealing a clear assembly switching during the presentation of a single stimulus (see lower panels of S 5 Fig).

Synchronized Event Transition Matrices and number of coactive cells for different network realizations

We present two different realizations of the numerical experiment performed in the sub-section *Physiological relevance for biological networks under different experimental conditions*. The difference between the realizations lies on the random connectivity matrix C_{ij}, which is generated at each realization with the same connection probability. The results are presented in S 7 Fig. More precisely, in S 7(a,e) Figs are reported the SETMs for maximal Q_0 ($g = 8$ for the chosen parameters). These are characterized by a large variability in their elements when compared with the corresponding SETMs obtained for decreased inhibition (namely, $g = 1$), shown in S 7(c,g) Figs, is always smaller. The difference between the two regimes is also evidenced in the number of coactive cells: at maximal Q_0 each state is well defined, as illustrated in S 7(b,f) Figs. Since diagonal elements (representing the number of neurons active in a given state) present larger bars compared with the off-diagonal ones (representing the overlap between two different states) Instead, in the set-up with $g = 1$ the states are hardly distinguishable, diagonal and off-diagonal bars have similar heights (as shown in S 7(d,h) Figs)

---

[1] Angulo-Garcia, D. and Torcini, A. (2014). Stable chaos in fluctuation driven neural circuits. *Chaos, Solitons & Fractals*, 69(0):233 – 245.

[2] Benettin, G., Galgani, L., Giorgilli, A., and Strelcyn, J.-M. (1980). Lyapunov characteristic exponents for smooth dynamical systems and for hamiltonian systems; a method for computing all of them. part 1: Theory. *Meccanica*, 15(1):9–20.

[3] Jahnke, S., Memmesheimer, R.-M., and Timme, M. (2009). How chaotic is the balanced state? *Front. Comp. Neurosci.*, 3(13).

[4] Monteforte, M. and Wolf, F. (2012). Dynamic flux tubes form reservoirs of stability in neuronal circuits. *Phys. Rev. X*, 2:041007.

[5] Politi, A. and Torcini, A. (2010). Stable chaos. In *Nonlinear Dynamics and Chaos: Advances and Perspectives*, pages 103–129. Springer.

[6] Ponzi, A. and Wickens, J. (2012). Input dependent cell assembly dynamics in a model of the striatal medium spiny neuron network. *Frontiers in systems neuroscience*, 6.

[7] Zillmer, R., Livi, R., Politi, A., and Torcini, A. (2006). Desynchronization in diluted neural networks. *Phys. Rev. E*, 74(3):036203.
