# Supplementary material for: Cell Assembly Dynamics of Sparsely-Connected Inhibitory Networks: A Simple Model for the Collective Activity of Striatal Projection Neurons
Source: PLoS Comput Biol. 2016 Feb 25;12(2):e1004778. doi: 10.1371/journal.pcbi.1004778 (PMC4767417; doi:10.1371/journal.pcbi.1004778)
Supplement: S1 Fig — Fraction of active neurons n* vs the synaptic strength, for several threshold definitions. A neuron is considered silent whenever it does not spike at least SΘ-times during the observation time. Panel a) for ΔV = 1 mV and b) for ΔV = 5 mV. The system is left to evolve during 107 spikes, after discarding 105 spike events of transient. Other parameters used in the simulation: K = 20, N = 400 and τα = 20ms. (PDF) [file pcbi.1004778.s002.pdf]

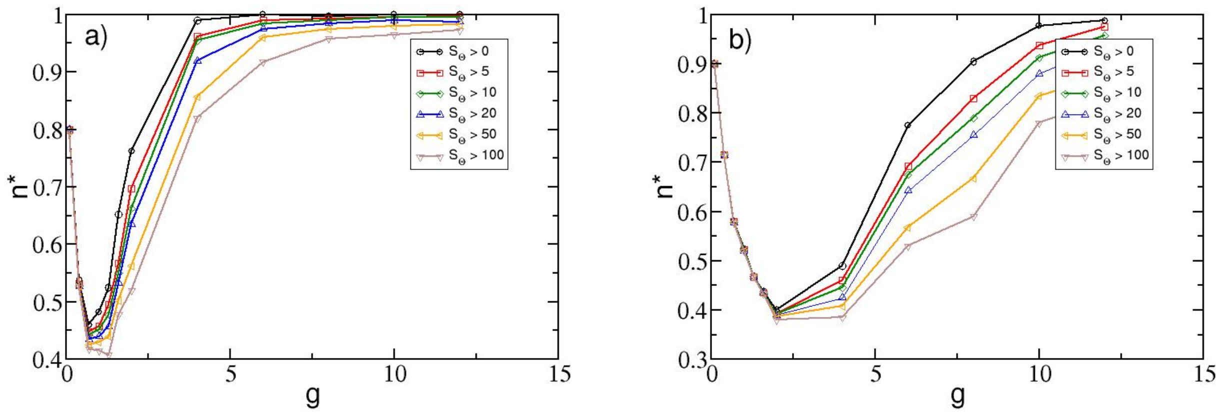

S 1. Fig. **Dependence of the value** $n*$ **on the chosen threshold** S_Θ Fraction of active neurons n^* vs the synaptic strength, for several threshold definitions. A neuron is considered silent whenever it does not spike at least S_Θ-times during the observation time. Panel a) for $\Delta V = 1$ mV and b) for $\Delta V = 5$ mV. The system is left to evolve during 10^7 spikes, after discarding 10^5 spike events of transient. Other parameters used in the simulation: $K = 20$, $N = 400$ and $\tau_\alpha = 20$ms.
