# Supplementary material for: Cell Assembly Dynamics of Sparsely-Connected Inhibitory Networks: A Simple Model for the Collective Activity of Striatal Projection Neurons
Source: PLoS Comput Biol. 2016 Feb 25;12(2):e1004778. doi: 10.1371/journal.pcbi.1004778 (PMC4767417; doi:10.1371/journal.pcbi.1004778)
Supplement: S2 Fig — Neuronal distributions of the average ISI¯ (a,d), of the coefficient of variation CV (b,e) and of the average effective synaptic input W¯i (c,f). The data in the first row are for ΔV = 1 mV and in the second one for ΔV = 5 mV. For ΔV = 1 mV (ΔV = 5 mV) black solid line with filled circles correspond to g = 1 (g = 4) and red dashed lines with open squares to g = 4 (g = 10). Insets of (a,d) and (c,f), same as the main figure in a lower g regime: g = 0.4 (g = 1) for ΔV = 1 mV (ΔV = 5 mV). The system is left to evolve during 107 spikes, after discarding 105 spike events. Other parameters used in the simulation: K = 20, N = 400 and τα = 20 ms. (PDF) [file pcbi.1004778.s003.pdf]

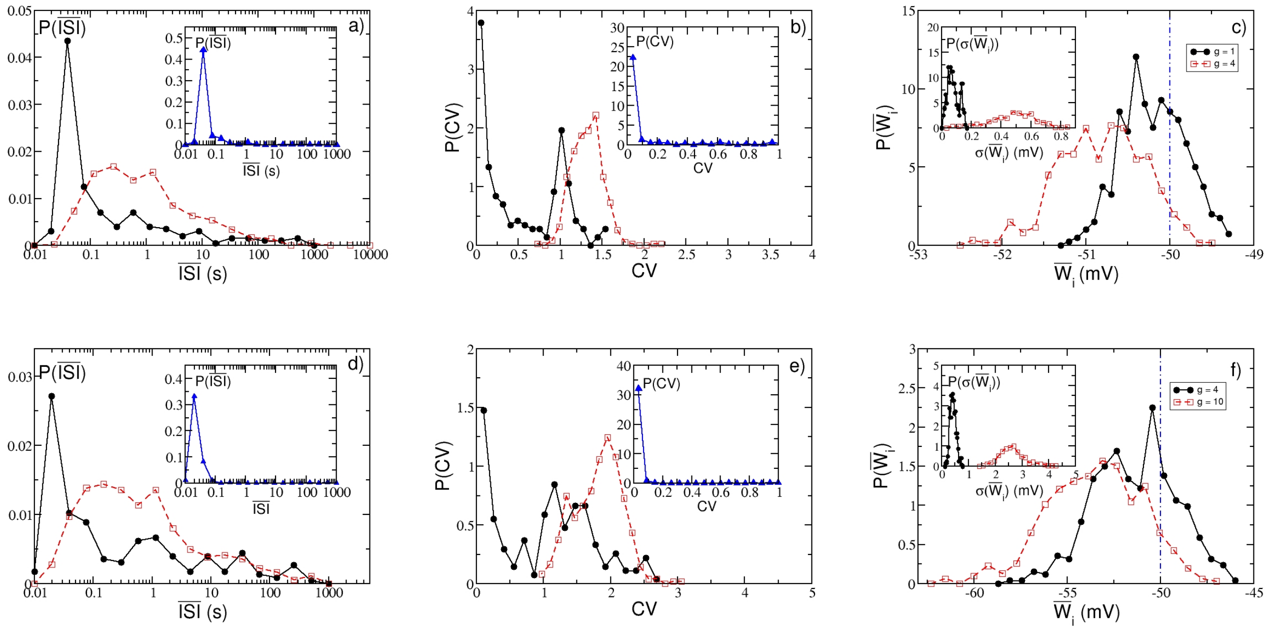

S 2. Fig. **Neuronal statistics.** Neuronal distributions of the average $\overline{ISI}$ (a,d), of the coefficient of variation CV (b,e) and of the average effective synaptic input $\overline{W}_i$ (c,f). The data in the first row are for $\Delta V = 1$ mV and in the second one for $\Delta V = 5$ mV. For $\Delta V = 1$ mV ($\Delta V = 5$ mV) black solid line with filled circles correspond to $g = 1$ ($g = 4$) and red dashed lines with open squares to $g = 4$ ($g = 10$). Insets of (a,d) and (c,f), same as the main figure in a lower g regime: $g = 0.4$ ($g = 1$) for $\Delta V = 1$ mV ($\Delta V = 5$ mV). The system is left to evolve during 10^7 spikes, after discarding 10^5 spike events. Other parameters used in the simulation: $K = 20$, $N = 400$ and $\tau_\alpha = 20$ ms.
