# Supplementary material for: Cell Assembly Dynamics of Sparsely-Connected Inhibitory Networks: A Simple Model for the Collective Activity of Striatal Projection Neurons
Source: PLoS Comput Biol. 2016 Feb 25;12(2):e1004778. doi: 10.1371/journal.pcbi.1004778 (PMC4767417; doi:10.1371/journal.pcbi.1004778)
Supplement: S3 Fig — a) Maximal Lyapunov exponent λ at a fixed τα = 20, as a function of the synaptic strength for ΔV = 1 mV (continuous line, filled circles) and ΔV = 5 mV (dashed line, empty squares). b) Maximal Lyapunov exponent λ as a function of the pulse duration τα for the parameters {ΔV,g} = {1 mV,4} (continuous line with filled circles) and {5 mV,8} (dashed line with empty squares). In both panels, the blue filled square indicates the triad {ΔV,g,τα} = {5 mV,8,20 ms}, and the red filled circle to {ΔV,g,τα} = {1 mV,4,20 ms}; these values are associated to the maximum values of Q0 obtained for excitability distributions with fixed width ΔV. The tangent space Eq (S17) is evolved during a period corresponding to 106 spikes, after discarding a transient of 105 spikes. Other parameters used in the simulation: K = 20, N = 400. (PDF) [file pcbi.1004778.s004.pdf]

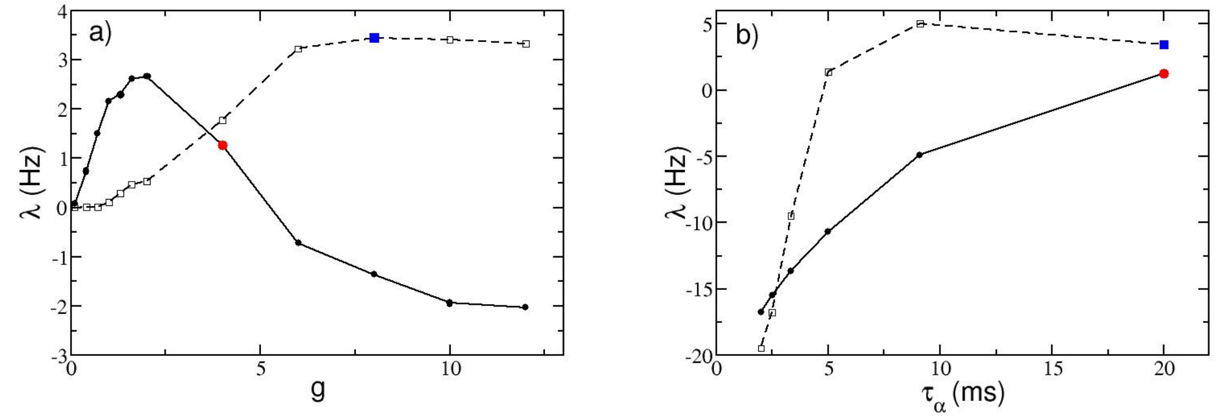

S 3. Fig. **Linear stability analysis**. a) Maximal Lyapunov exponent λ at a fixed $\tau_\alpha = 20$, as a function of the synaptic strength for $\Delta V = 1$ mV (continuous line, filled circles) and $\Delta V = 5$ mV (dashed line, empty squares). b) Maximal Lyapunov exponent λ as a function of the pulse duration τ_α for the parameters $\{\Delta V, g\} = \{1 \text{ mV}, 4\}$ (continuous line with filled circles) and $\{5 \text{ mV}, 8\}$ (dashed line with empty squares). In both panels, the blue filled square indicates the triad $\{\Delta V, g, \tau_\alpha\} = \{5 \text{ mV}, 8, 20 \text{ ms}\}$, and the red filled circle to $\{\Delta V, g, \tau_\alpha\} = \{1 \text{ mV}, 4, 20 \text{ ms}\}$; these values are associated to the maximum values of Q_0 obtained for excitability distributions with fixed width ΔV. The tangent space (S1-S3 Eqs) is evolved during a period corresponding to 10^6 spikes, after discarding a transient of 10^5 spikes. Other parameters used in the simulation: $K = 20$, $N = 400$.
