# Supplementary material for: Cell Assembly Dynamics of Sparsely-Connected Inhibitory Networks: A Simple Model for the Collective Activity of Striatal Projection Neurons
Source: PLoS Comput Biol. 2016 Feb 25;12(2):e1004778. doi: 10.1371/journal.pcbi.1004778 (PMC4767417; doi:10.1371/journal.pcbi.1004778)
Supplement: S4 Fig — a) Metrics entering in the definition of Q0 and their dependence from τα. From top to bottom: Averaged coefficient of variation 〈CV〉N, standard deviation of the cross-correlation matrix σ(C), and the fraction of active neurons n*. b) ΔMd as a function of τα. The system is left to evolve during 107 spikes, after discarding 105 transient spike events. Parameters here used ΔV = 5 mV, g = 8, K = 20, N = 400. (PDF) [file pcbi.1004778.s005.pdf]

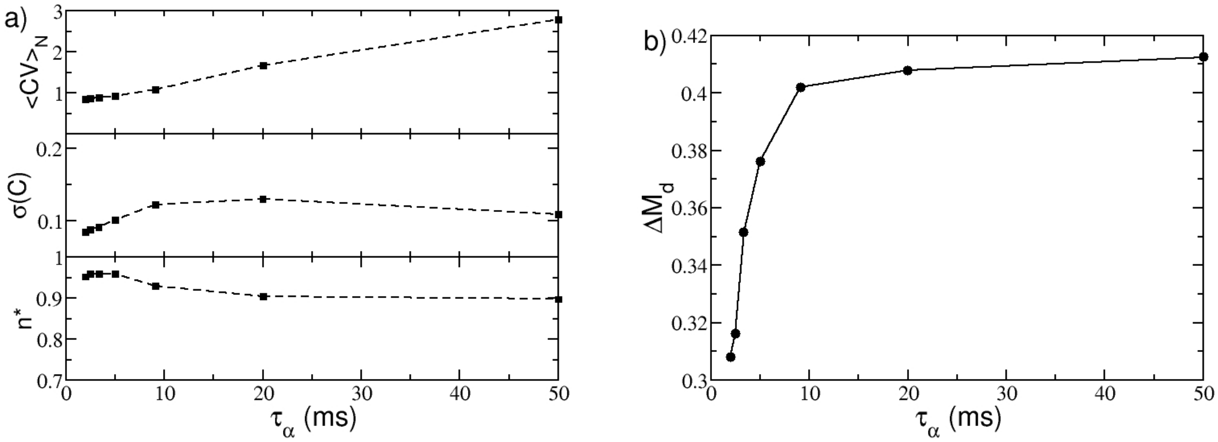

S 4. Fig. **Metrics of the structured activity vs synaptic time decay.** a) Metrics entering in the definition of Q_0 and their dependence from τ_α. From top to bottom: Averaged coefficient of variation $\langle CV \rangle_N$, standard deviation of the cross-correlation matrix $\sigma(C)$, and the fraction of active neurons n^*. b) ΔM_d as a function of τ_α. The system is left to evolve during 10^7 spikes, after discarding 10^5 transient spike events. Parameters here used $\Delta V = 5$ mV, $g = 8$, $K = 20$, $N = 400$.
