# Supplementary material for: Cell Assembly Dynamics of Sparsely-Connected Inhibitory Networks: A Simple Model for the Collective Activity of Striatal Projection Neurons
Source: PLoS Comput Biol. 2016 Feb 25;12(2):e1004778. doi: 10.1371/journal.pcbi.1004778 (PMC4767417; doi:10.1371/journal.pcbi.1004778)
Supplement: S5 Fig — Upper panel, another realization of the network with the same parameters as chosen in the main text and τα = 20ms. Lower panel, network with τα = 2ms. From left to right it is depicted the raster plot colored according to the k-means algorithm with k = 25, vertical lines indicates the change of the presented stimulus. In the middle column is reported the state transition matrix calculated over a time span of 20 seconds (the stimulation protocol is repeated 5 times). Rightmost column reports the state transition matrix for a block 4 s × 4 s averaged over r = 5 successive presentations of the inputs. (PDF) [file pcbi.1004778.s006.pdf]

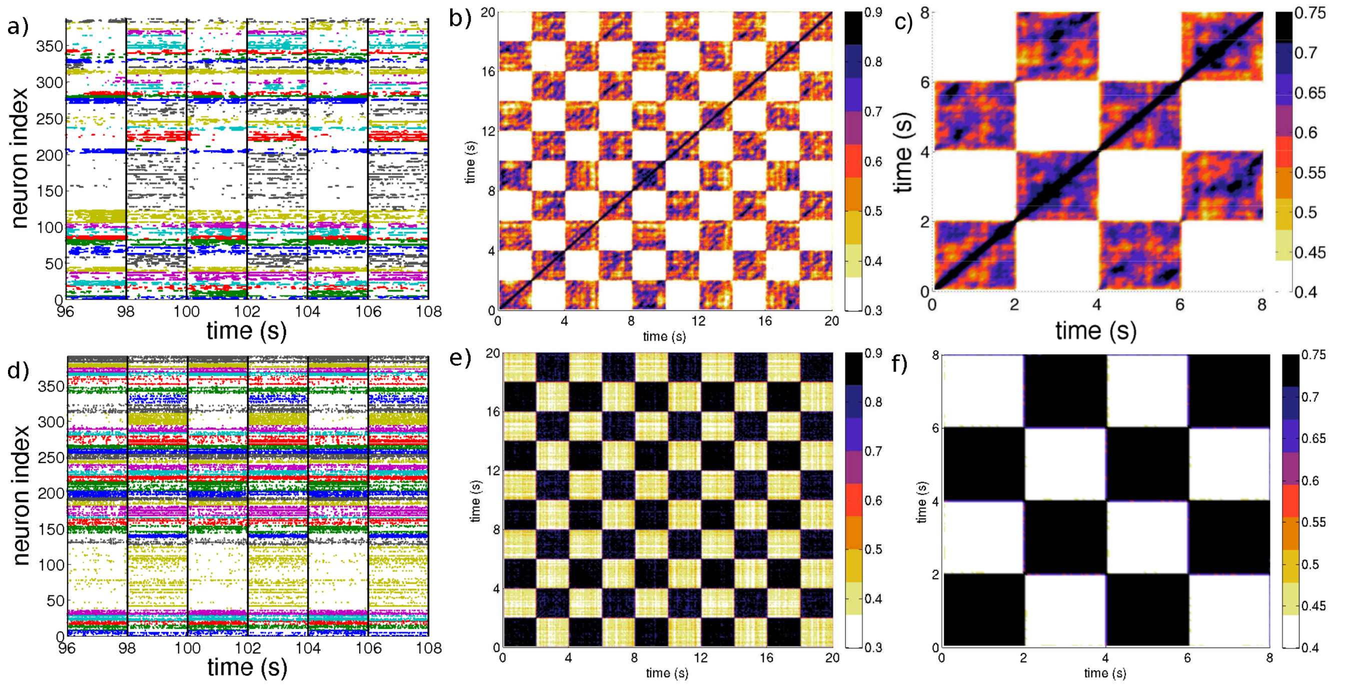

S 5. Fig. **Two stimuli presentation.** Upper panel, another realization of the network with the same parameters as chosen in the main text and $\tau_\alpha = 20$ms. Lower panel, network with $\tau_\alpha = 2$ms. From left to right it is depicted the raster plot colored according to the *k-means* algorithm with k=25, vertical lines indicates the change of the presented stimulus. In the middle column is reported the state transition matrix calculated over a time span of 20 seconds (the stimulation protocol is repeated 5 times). Rightmost column reports the state transition matrix for a block 4 s $\times$ 4 s averaged over $r = 5$ successive presentations of the inputs.
