# Supplementary material for: Cell Assembly Dynamics of Sparsely-Connected Inhibitory Networks: A Simple Model for the Collective Activity of Striatal Projection Neurons
Source: PLoS Comput Biol. 2016 Feb 25;12(2):e1004778. doi: 10.1371/journal.pcbi.1004778 (PMC4767417; doi:10.1371/journal.pcbi.1004778)
Supplement: S6 Fig — Dissimilarity measure in time for an observation window of length a) TE = 2 s and b) TE = 10 s, for two values of τα = 20 ms (black circles) and τα = 2 ms (red squares) at a fixed value of f = 0.2. It is clearly observed that τα = 20 ms more effectively differentiates the similar inputs in both observation windows, as seen by the larger values of dissimilarity respect to the τα = 2ms. The initial increase of df(t) observable for τα = 20 ms in panel (a) is probably due to the fact that the dynamics for this choice of parameters is chaotic as shown in the Linear stability analysis sub-section. Therefore the increase can be associated to a transient evolution towards the final attractor. Other parameters used: ΔT = 50 ms, g = 8, N = 400, K = 20. (PDF) [file pcbi.1004778.s007.pdf]

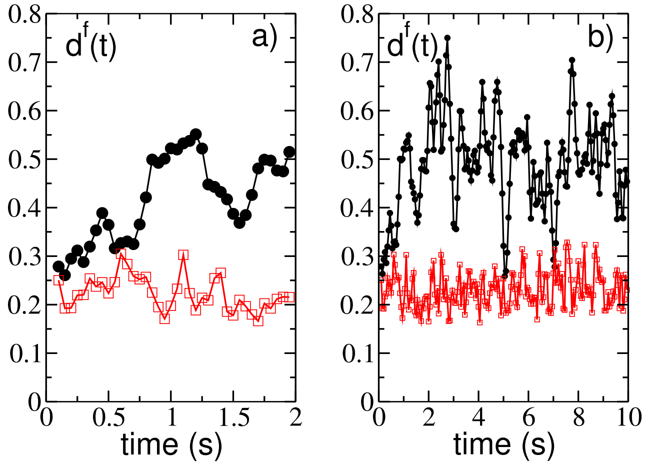

S 6. Fig. **Pattern Separation.** Dissimilarity measure in time for an observation window of length a) $T_E = 2$ s and b) $T_E = 10$ s, for two values of τ_α=20 ms (black circles) and τ_α=2 ms (red squares) at a fixed value of $f = 0.2$. It is clearly observed that $\tau_\alpha = 20$ ms more effectively differentiates the similar inputs in both observation windows, as seen by the larger values of dissimilarity respect to the $\tau_\alpha = 2$ms. The initial increase of $d^f(t)$ observable for $\tau_\alpha = 20$ ms in panel (a) is probably due to the fact that the dynamics for this choice of parameters is chaotic as shown in the *Linear stability analysis* sub-section. Therefore the increase can be associated to a transient evolution towards the final attractor. Other parameters used: $\Delta T = 50$ ms, $g = 8$, $N = 400$, $K = 20$.
