# Supplementary material for: Cell Assembly Dynamics of Sparsely-Connected Inhibitory Networks: A Simple Model for the Collective Activity of Striatal Projection Neurons
Source: PLoS Comput Biol. 2016 Feb 25;12(2):e1004778. doi: 10.1371/journal.pcbi.1004778 (PMC4767417; doi:10.1371/journal.pcbi.1004778)
Supplement: S7 Fig — Two different realizations of the numerical experiment reported in the subsection Physiological relevance for biological networks under different experimental conditions of the main text. First (third) column reports two realizations of the SETM estimated for g = 8 (g = 1). Second (fourth) column displays the number of coactive cells in the corresponding cases for g = 8 (g = 1). The other parameters for the reported simulations are ΔV = 5 mV, K = 20, N = 400. (PDF) [file pcbi.1004778.s008.pdf]

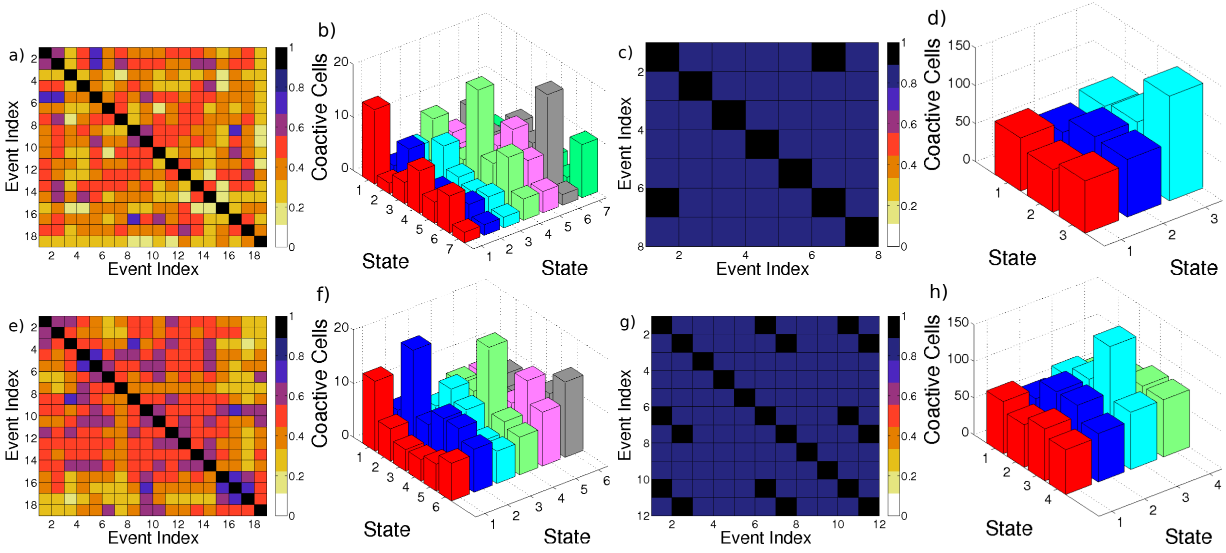

S 7. Fig. **Response of the network to an increase in the excitability.** Two different realizations of the numerical experiment reported in the subsection *Physiological relevance for biological networks under different experimental conditions* of the main text. First (third) column reports two realizations of the SETM estimated for $g = 8$ ($g = 1$). Second (fourth) column displays the number of coactive cells in the corresponding cases for $g = 8$ ($g = 1$). The other parameters for the reported simulations are $\Delta V = 5$ mV, $K = 20$, $N = 400$.
